# Corrosion inhibition of mild steel in sulfuric acid solution by loquat (Eriobotrya japonica Lindl.) leaves extract

**DOI:** 10.1038/s41598-018-27257-9

**Published:** 2018-06-14

**Authors:** Xingwen Zheng, Min Gong, Qiang Li, Lei Guo

**Affiliations:** 1 School of Chemical and Environmental Engineering, Sichuan University of Science & Engineering, Zigong, 643000 China; 2 Key Laboratory of Material Corrosion and Protection of Sichuan Province, Zigong, 643000 China; 3 School of Materials and Chemical Engineering, Tongren University, Tongren, 554300 China

## Abstract

The inhibition performance and mechanism of loquat leaves extract (LLE) for the corrosion of mild steel in 0.5 M H_2_SO_4_ were investigated using weight loss method, electrochemical measurements and scanning electron microscope (SEM). The results revealed that LLE acted as a modest cathodic inhibitor, its inhibition efficiency increased with the concentration of LLE and reached a maximum value of 96% at the 100% V/V concentration, but decreased with incremental temperature. Besides, it was found that the adsorption of LLE on steel surface obeyed Langmuir adsorption isotherm, and then the thermodynamic and kinetic parameters were further determined accordingly. Furthermore, LLE was preliminarily separated by pH-gradient sedimentation and the synergistic inhibition between the isolates was investigated.

## Introduction

In recent years, plant extracts as corrosion inhibitors have attracted extensive attention due to their properties of environment-friendliness, low cost and renewability^1–3^. Notably, the first patented corrosion inhibitors applied for restraining iron corrosion in acid media are either natural products such as flour, yeast etc., or byproducts of food industries^4–7^. Recently, hundreds of plant extracts have been reported as inhibitors for steel in acid solutions^1–3,7–32^. For instance, Li *et al*.^1,8,9^ studied the corrosion inhibition of steel by eco-friendly bamboo leaves extract in HCl, H_2_SO_4_, H_3_PO_4_, and citric acid solution. Lebrini *et al*.^17,26^ noticed of steel corrosion inhibition in hydrochloric acid medium by alkaloids extracted from Oxandra asbeckii plant as well as Aniba rosaeodora plant. In addition, plant extracts have also been reportedly used as corrosion inhibitors for other metal and alloy, including Aloe vera^33^ and Mansoa alliacea^34^ for zinc, Mimosa^35^ and Camellia sinensis^36^ for brass, Morinda tinctoria^37^ and Myrtus communis^38^ for copper, and Spondias mombin L.,^39^ Jasminum nudiflorum Lindl.,^40^ Gossipium hirsutum L.^41^ and Date palm^42^ for aluminum. However, compared with the huge number of plant resources and the potential demand for efficient green corrosion inhibitors, more research is still needed. And, the phytochemical investigations to the effective inhibitive composition of plant extract are still scarce, which has important significance for understanding the inhibition mechanism of plant extract and the development of new inhibitors.

Eriobotrya japonica Lindl. (loquat) is a flowering plant belonging to the Rosaceae family and widely distributed in East Asia including China, Korea, Japan and many other countries^43–45^. This paper reports the effect of acid extract of loquat leaves as an environmentally friendly corrosion inhibitor for mild steel in 0.5 M H_2_SO_4_ using gravimetric technique, potentiodynamic polarization, electrochemical impedance spectroscopy (EIS) and microscopic examination. Moreover, LLE was preliminarily separated by pH-gradient sedimentation, thus the isolates were characterized through high performance liquid chromatograph (HPLC), mass spectrometry (MS), amino acid analyzer and Fourier transform infrared spectroscopy (FTIR), and the synergistic inhibition between the isolates was investigated.

## Results and Discussion

**Figure 1:**
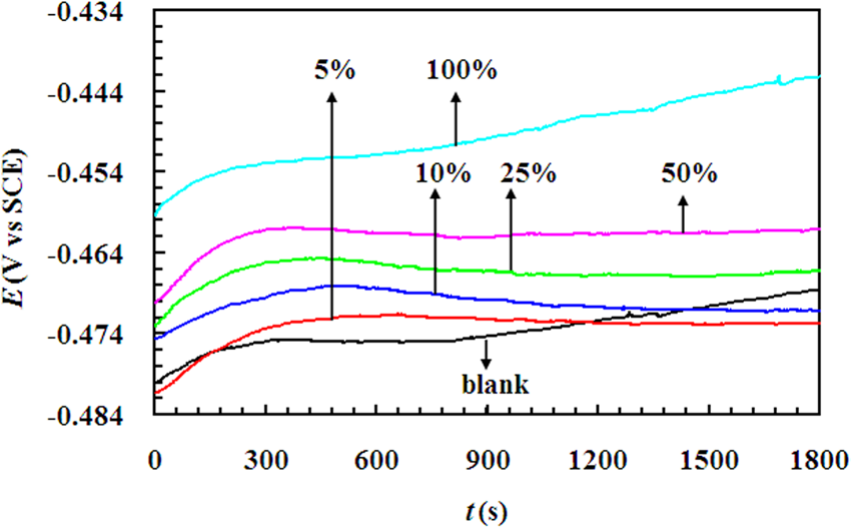
The OCP-time curves for mild steel in 0.5 M H_2_SO_4_ solution containing different different V/V concentrations of LLE at 298 K.

The curves of open circuit potential (OCP) versus time are depicted in Figure 1, it could be seen that the OCP of steel electrode in the studied solutions nearly reached a steady level after immersion for 30 minutes, and the values of OCP, taking the average of the last 600 seconds, were −0.4706, −0.4728, −0.4711, −0.4667, −0.4615 and −0.4446 V vs. SCE for LLE V/V concentrations of 0, 5, 10, 25, 50 and 100%, respectively.

**Figure 2:**
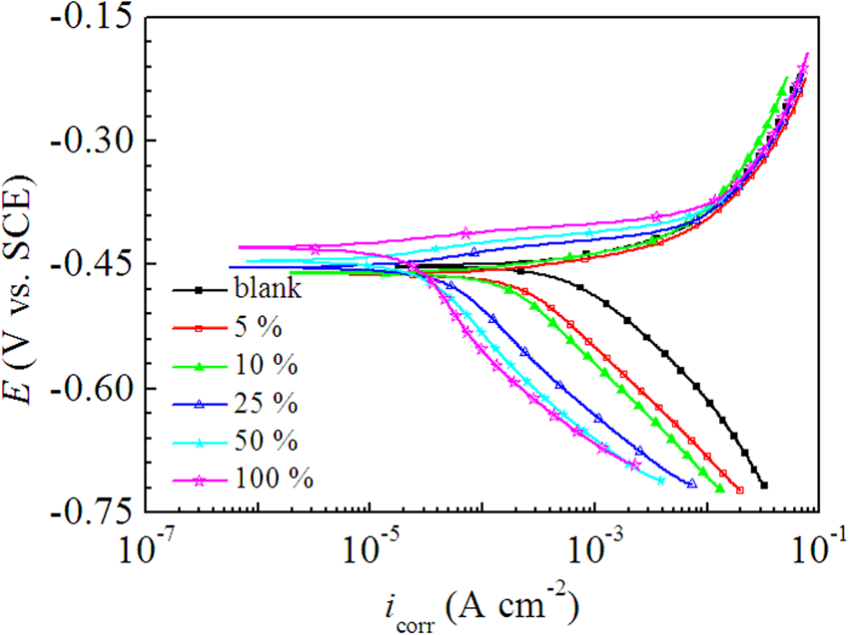
Potentiodynamic polarization curves of mild steel in 0.5 M H_2_SO_4_ solutions without and with different V/V concentrations of LLE at 298 K.

Figure 2 illustrates the potentiodynamic polarization curves of mild steel in 0.5 M H_2_SO_4_ solutions containing different concentrations of LLE at 298 K. As shown in Figure 2, since there was no linear Tafel region on anodic polarization curve, thus the electrochemical parameters, including corrosion potential (*E*_corr_), corrosion current density (*I*_corr_), cathodic Tafel slope (*β*_c_) and inhibition efficiency (*η*), were determined by extrapolation of the cathodic part of the polarization curve to *E*_corr_, and listed in Table 1. Figure 2 and Table 1 show that the presence of LLE caused a remarkable decrease of *I*_corr_ value, and a higher concentration of LLE resulted in an even lower *I*_corr_ value. Accordingly, the inhibition efficiency increased along with the increase of LLE concentration, and the maximum inhibition efficiency reached 96% at the 100% V/V concentration. Moreover, Figure 2 also indicates that the addition of LLE prominently shifted the cathodic branch of polarization curves to lower values of current densities, suggesting strong inhibitive effect of LLE on the cathodic reduction of hydrogen ions. Besides, it can be observed from in Figure 2 that the cathodic polarization curves were almost parallel to each other, the values of *β*_c_ in Table 1 exhibited small fluctuations accordingly, which indicate that LLE dose not impact the mechanism of hydrogen reduction and the hydrogen evolution is activation-controlled^46^. However, the anodic polarization curve with inhibitor almost overlapped with that in the blank solution in the strong polarization region, despite small decrease of anodic current density at low polarization potential, which is attributed to the desorption of inhibitors. Besides, the values of *E*_corr_ did not vary significantly with a displacement less than 30 mV (as seen in Table 1). Overall, LLE can be seen as a modest cathodic inhibitor^47,48^.

**Table 1:** The electrochemical parameters for mild steel in 0.5 mol/L H_2_SO_4_ solution without and with different V/V concentrations of LLE at 298 K.

*c*(% V/V)	*E*_corr_ (V vs. SCE)	*I*_corr_(mA cm^−2^)	−*β*_c_(mV dec^−1^)	*η*(%)
blank	−0.452	0.578	123.1	\
5	−0.462	0.202	126.6	65.0
10	−0.460	0.145	130.7	74.9
25	−0.453	0.043	138.6	92.5
50	−0.446	0.028	157.1	95.2
100	−0.429	0.021	192.7	96.3

**Figure 3:**
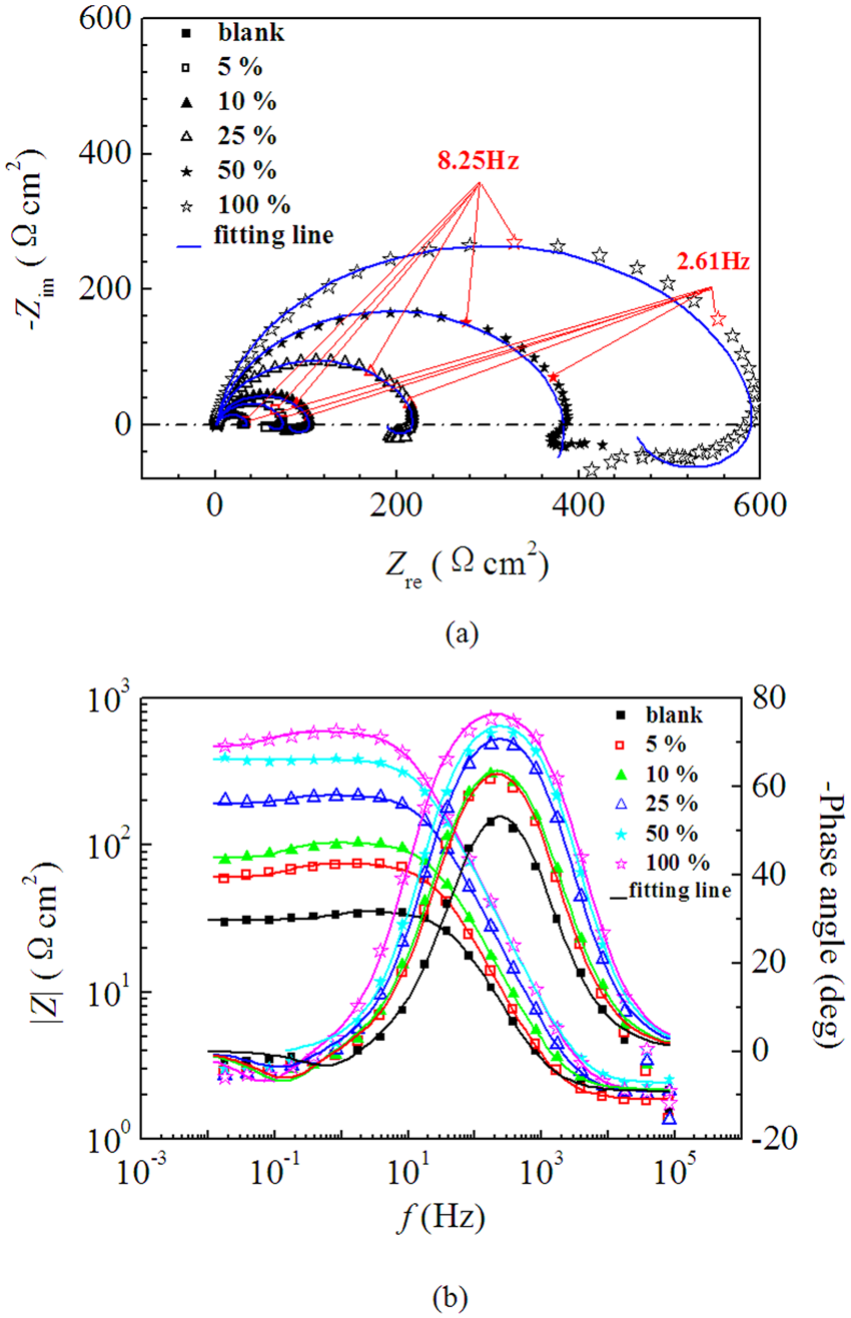
Nyquist plots (**a**) and Bode plots (**b**) for mild steel in 0.5 M H_2_SO_4_ solution without and with different V/V concentrations of LLE at 298 K.

Figure 3 shows the Nyquist plots and Bode plots for mild steel in 0.5 M H_2_SO_4_ solution without and with different concentrations of LLE at 298 K. It can be seen that the addition of LLE did not change the profile of Nyquist plots, which consist of a capacitive loop at high frequencies (HF) and an inductive loop at low frequencies (LF), suggesting that the presence of the LLE has little influence on the corrosion mechanism^1,11^. The HF capacitive loop is usually related to the charge transfer of the corrosion process and the resultant double layer behavior^49–51^. However, due to the frequency dispersion as a result of the roughness and non-homogeneity of electrode surface^52^, the capacitive loop exhibits a depressed semicircle. Meanwhile, the LF inductive loop is attributed to the relaxation process caused by adsorption of species such as (SO_4_^2−^)_ads_ and (H^+^)_ads_^48,51^. Figure 3b shows the impedance data in the Bode plots of impedance magnitude (|Z|) and phase angle over the whole frequency range obtained for the mild steel electrode with LLE V/V concentrations between 0 and 100%. The phase angles of the uninhibited and inhibited solutions are 52.3°, 61.6°, 62.8°, 69.9°, 72.9° and 75.7°, respectively, the values of phase angle and impedance magnitude at LF increase with the increase of LLE concentration, which indicates better protection behavior of LLE with higher concentrations.

**Figure 4:**
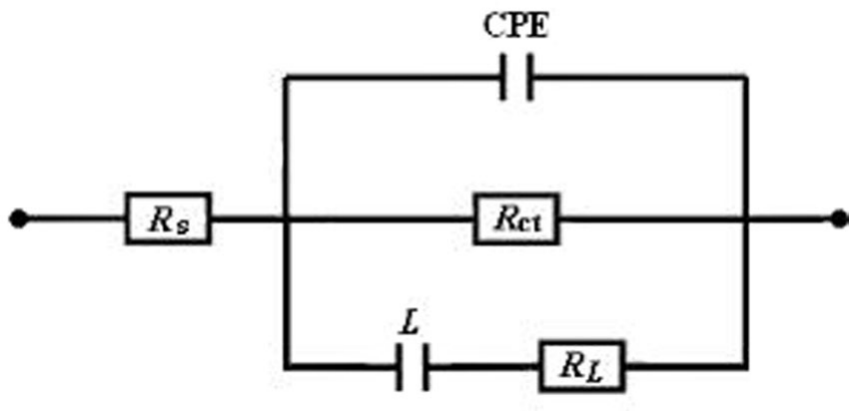
Equivalent circuits used to fit the EIS.

The EIS data are simulated with the equivalent circuits as shown in Figure 4 using the ZSimpWin software and the impedance parameters are given in Table 2. In the circuits, *R*_s_, *R*_ct_ and *R*_*L*_ represent the solution resistance, charge transfer resistance and inductive resistance, respectively, *L* is the inductive elements, and constant phase element (CPE) is used to replace a double layer capacitance in order to obtain a better fitting. The impedance of CPE can be described as follows^52,53^:

1 $${Z}_{{\rm{CPE}}}=\tfrac{1}{{{Y}}_{0}{(j{\omega })}^{{n}}}$$

where *Y*_0_ is the CPE constant, *j* is the imaginary unit, *ω* is the angular frequency, and *n* is the deviation parameter, has the meaning of the phase shift. In addition, the double layer capacitance (*C*_dl_) and inhibition efficiency (*η*) are calculated according to the following equations^52,53^:

2 $${{C}}_{{dl}}={{Y}}_{0}{({\omega })}^{{n}-1}={{Y}}_{0}{(2{\pi }{{f}}_{{{z}}_{im}-Max})}^{{n}-1}$$

3 $${\eta }=\frac{{{R}}_{{\rm{ct}}}-{{R}}_{{\rm{ct}},0}}{{{R}}_{{\rm{ct}}}}\times 100$$

where *ω* is the angular frequency at the maximum value of the imaginary part (*Z*_im-Max_) of the impedance spectrum^52^, *R*_ct,0_ and *R*_ct_ are the charge transfer resistances without and with inhibitor, respectively.

**Table 2:** The EIS parameters for mild steel in 0.5 mol/L H_2_SO_4_ solution without and with different V/V concentrations of LLE at 298 K.

*C* (% V/V)	*R*_s_ (Ω cm^2^)	*Y*_0_ × 10^−6^ (S s^n^ cm^−2^)	n	*R*_ct_ (Ω cm^2^)	*C*_dl_ (μF cm^−2^)	*L* (Ω cm^2^)	*R*_*L*_ (Ω cm^2^)	*η* (%)
blank	2.1	202.0	0.87	34	96.6	46	193	\
5	1.9	143.1	0.89	74	81.7	323	276	54.1
10	2.2	117.4	0.88	103	65.1	429	343	67.0
25	2.1	62.7	0.91	219	41.0	1741	1359	84.5
50	2.4	40.5	0.92	384	28.2	3124	2624	91.1
100	2.1	37.9	0.92	596	27.6	4850	2023	94.3

As shown in Table 2, the *R*_ct_ values increased while the values of *C*_dl_ declined when increasing LLE concentration, correspondingly, the inhibition efficiency increased with the increase of the concentration of LLE, which indicate that the inhibitor molecules adsorbed at the metal/solution interface as a barrier layer, effectively protecting the mild steel from aggressive attack by the acid solution. The values of *n* became closer to one as the increase of LLE concentration, indicating the metal surface become more homogeneous due to the adsorption of inhibitor molecules^1^.

The results of weight loss measurements for the corrosion of mild steel in 0.5 M H_2_SO_4_ solution without and with different concentrations of LLE for 4 h at different temperatures are given in Table 3. It is clear that the corrosion rate decreases under the same temperature when increasing inhibitors concentration, while it increases with rising of temperature, additionally, the inhibition efficiency at same concentration of inhibitor also decreased at higher temperature. At the 100% V/V concentration, the inhibition efficiency of LLE reached maximum value of 96.2, 95.0, 89.5 and 89.2% for 298, 308, 318 and 328 K, respectively, which mean LLE is an effective inhibitor for the corrosion of mild steel in 0.5 M H_2_SO_4_ solution. In addition, the inhibition efficiencies obtained from weight loss measurements are in good agreement with those obtained by electrochemical measurements.

**Table 3:** Corrosion parameters obtained from weight loss measurements for mild steel in 0.5 M H_2_SO_4_ solution without and with different V/V concentrations of LLE for 4 h at different temperatures.

*T*(K)	*c*(% V/V)	*v*(g m^−2^ h^−1^)	*η*(%)	*θ*
298	blank	13.54	/	/
	5	3.86	71.5	0.715
	10	2.57	81.1	0.811
	25	1.98	85.4	0.854
	50	1.22	91.0	0.910
	100	0.52	96.2	0.962
308	blank	25.42	/	/
	5	7.72	69.6	0.696
	10	5.48	78.4	0.784
	25	3.93	84.6	0.845
	50	2.91	88.5	0.885
	100	1.28	95.0	0.950
318	blank	37.92	/	/
	5	16.05	57.7	0.577
	10	11.27	70.3	0.703
	25	9.10	76.0	0.760
	50	6.23	83.6	0.836
	100	3.98	89.5	0.895
328	blank	60.89	/	/
	5	40.65	33.2	0.332
	10	23.23	61.9	0.619
	25	16.20	73.4	0.734
	50	10.05	83.5	0.835
	100	6.57	89.2	0.892

Moreover, the inhibition efficiency of LLE is compared with that of other plant extract reported in literature^2,28,54–60^, the related data are listed in Table 4. It can be seen that the inhibition efficiency of LLE is slightly less than that of Radish seed extract at the V/V concentration of 10%, and compared with the maximum corrosion inhibition efficiency reported in the literature, the inhibition efficiency of LLE is close to that of Houttuynia cordata stem extract and Barley extract, slightly lower than that of Agetes erecta extract and Litchi peel extract, but higher than that of the extract of Medicago sativa, Anacyclus pyrethrum extract, Salvia aucheri mesatlantica, and so on, which suggest that LLE is an effective inhibitor.

**Table 4:** The inhibition efficiency of LLE on the corrosion of steel in 0.5 M H_2_SO_4_ solution compared with that of other extract described in literature.

Plant extract	*C* ^1^	*η*(%)^2^	*T*(K)	Reference
LLE	10% V/V	81.1/67.0/74.9	298	this work
Radish seed extract ^3^	10% V/V	−/80.2/79.3	303	^28^
LLE	100% V/V	96.2/94.3/96.3	298	this work
Medicago sativa leaf	500 ppm	50.0/ – /92.0	298	^54^
Houttuynia cordata stem	3.0 g/L	98.3/94.8/94.4	298	^55^
Anacyclus pyrethrum leaf and stem	350 mg/L	−/87.0/82.0	303	^56^
Anacyclus pyrethrum flower	350 mg/L	−/88.9/84.3	303	^56^
Anacyclus pyrethrum root	350 mg/L	−/79.3/79.9	303	^56^
Barley	0.84 g/L	−/94.2/94.6	303	^57^
Agetes erecta (Marigold flower)	1.0 g/L	96.3/98.1/98.2	303	^2^
Litchi (Litchi Chinensis) peels	3.0 g/L	−/97.8/95.7	298	^58^
Aerial part of Salvia aucheri mesatlantica	2.0 g/L	70.1/84.2/85.5	298 ^4^	^59^
Coconut coir dust	0.5 g/L	87.0/94.3/66.5	303	^60^

NOTE: ^1^Concentration corresponding to the maximum corrosion inhibition efficiency. ^2^The inhibition efficiency was listed in the order as follows: weight loss, electrochemical impedance spectroscopy and potentiodynamic polarization. ^1^The inhibition efficiency was tested in 1 M H_2_SO_4_ solution. ^4^The temperature of weight loss method is 303 K.

It is well known that the inhibition effect of organic inhibitor is based on their adsorption at the metal/solution interfaces, therefore, it is important to understand the adsorption behavior of inhibitor for revealing its inhibition mechanism. The adsorption isotherm can provide vital information regarding the interaction between the inhibitor and the metal surface. Thus, several adsorption isotherms are employed to fit the surface coverage (*θ*) obtained from weight loss measurements. Langmuir isotherm is found to provide the optimal description of the adsorption behavior of LLE on the mild steel surface, which can be expressed by the following equation^61,62^:

4 $$\frac{{c}}{{\theta }}=\frac{1}{{{K}}_{{\rm{ads}}}}+{c}$$

where *c* is the inhibitor concentration, *θ* is the surface coverage and *K*_ads_ is the adsorptive equilibrium constant, respectively.

**Figure 5:**
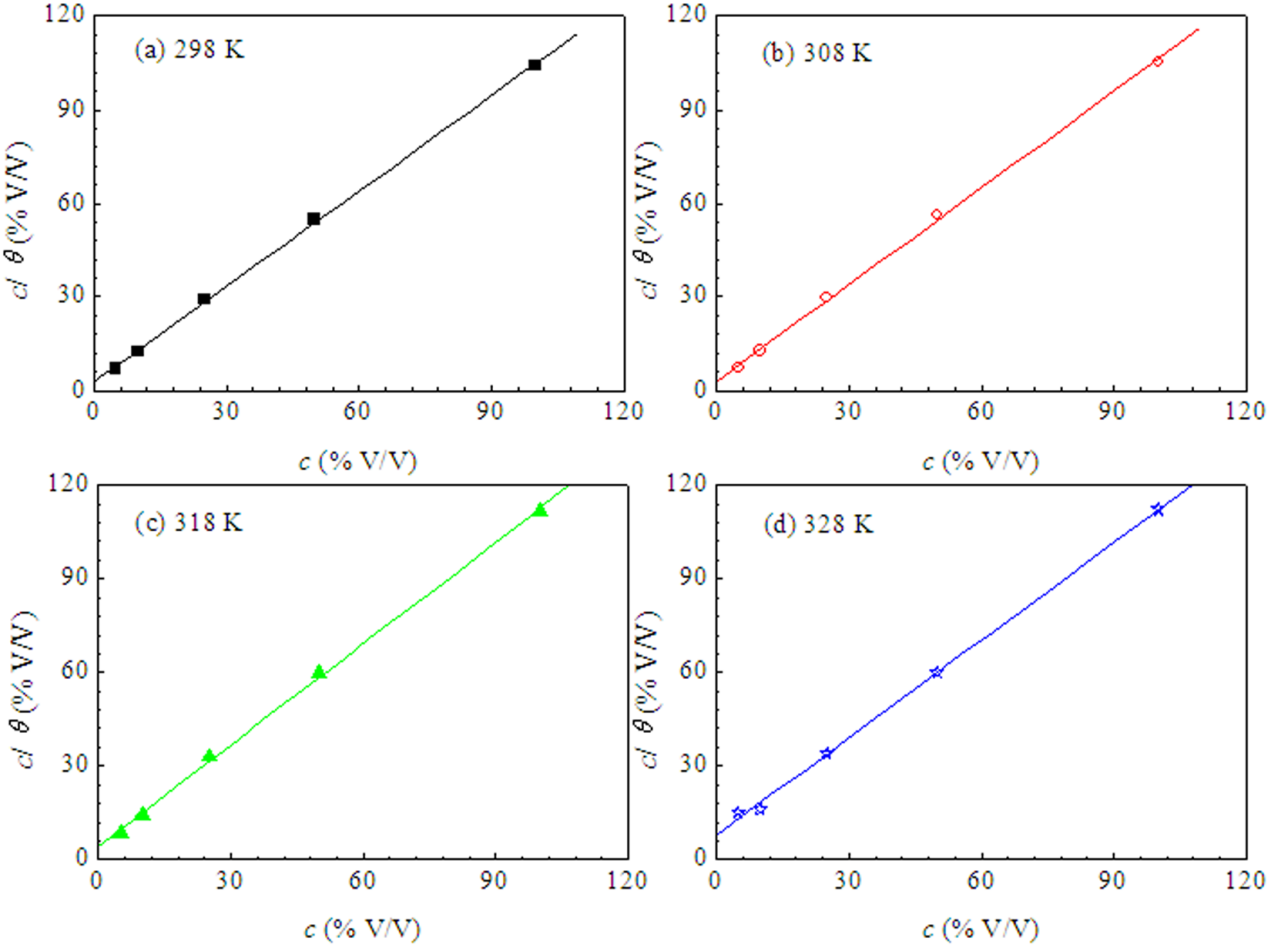
Langmuir isotherm plots for mild steel in 0.5 M H_2_SO_4_ solution containing different V/V concentrations of LLE at different temperature.

**Figure 6:**
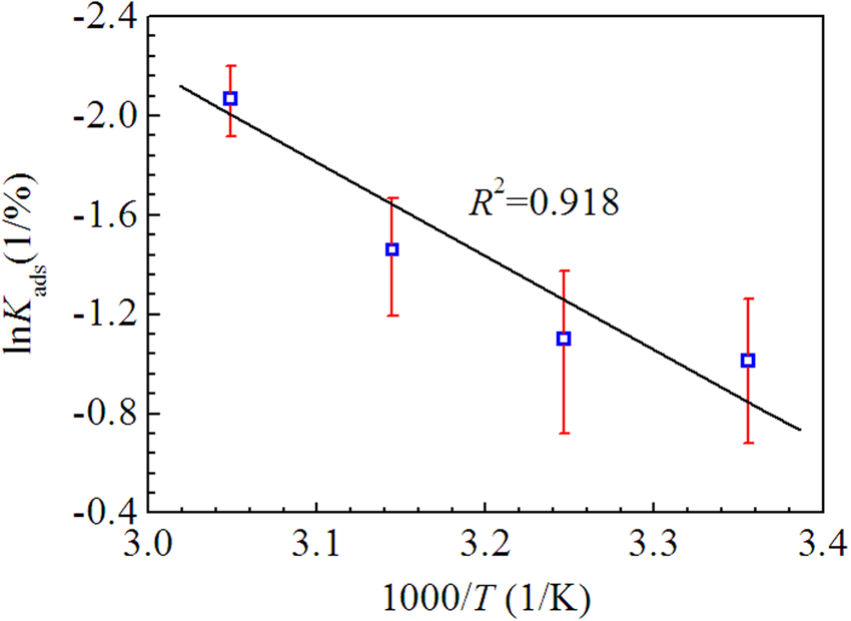
The relationship between ln*K*_ads_ and (1000/*T*) for mild steel in 0.5 M H_2_SO_4_ solution containing LLE.

The plots of *c*/*θ* versus *c* yield a straight line at different temperature as shown in Figure 5, confirming that the adsorption of LLE on the mild steel in 0.5 M H_2_SO_4_ solution obeys Langmuir isotherm. From the intercepts of the straight lines in Figure 5, the values of *K*_ads_ at different temperatures were calculated and listed in Table 5. The *K*_ads_ values decreased with the increase of temperature, which corresponded to the reduction of inhibition efficiency with rising temperature. Moreover, the adsorption enthalpy (${\rm{\Delta }}{H}_{{\rm{ads}}}^{0}$) can be calculated using Van’t Hoff equation^62^:

5 $$\mathrm{ln}\,{{K}}_{{\rm{ads}}}=-\frac{{\rm{\Delta }}{{H}}_{{\rm{ads}}}^{{\rm{0}}}}{{RT}}+{\rm{constant}}$$

where *R* is the universal gas constant, *T* is the thermodynamic temperature. Figure 6 shows that there is a linear relationship between ln*K*_ads_ and 1000/*T*, thus the value of ${\rm{\Delta }}{H}_{{\rm{ads}}}^{0}$ is calculated according to the Equation (5) and listed in Table 5, the negative value of ${\rm{\Delta }}{H}_{{\rm{ads}}}^{0}$ suggests the adsorption of the effective inhibitor component in LLE onto the mild steel surface is an exothermic process^62,63^.

**Table 5:** The thermodynamic parameters for mild steel in 0.5 M H_2_SO_4_ solution containing different V/V concentrations of LLE at different temperature.

*T*(K)	*K*_ads_(1/%)	${\rm{\Delta }}{H}_{{\rm{ads}}}^{0}$ (kJ/mol)
298	0.363	−31.34
308	0.333	
318	0.232	
328	0.127

**Figure 7:**
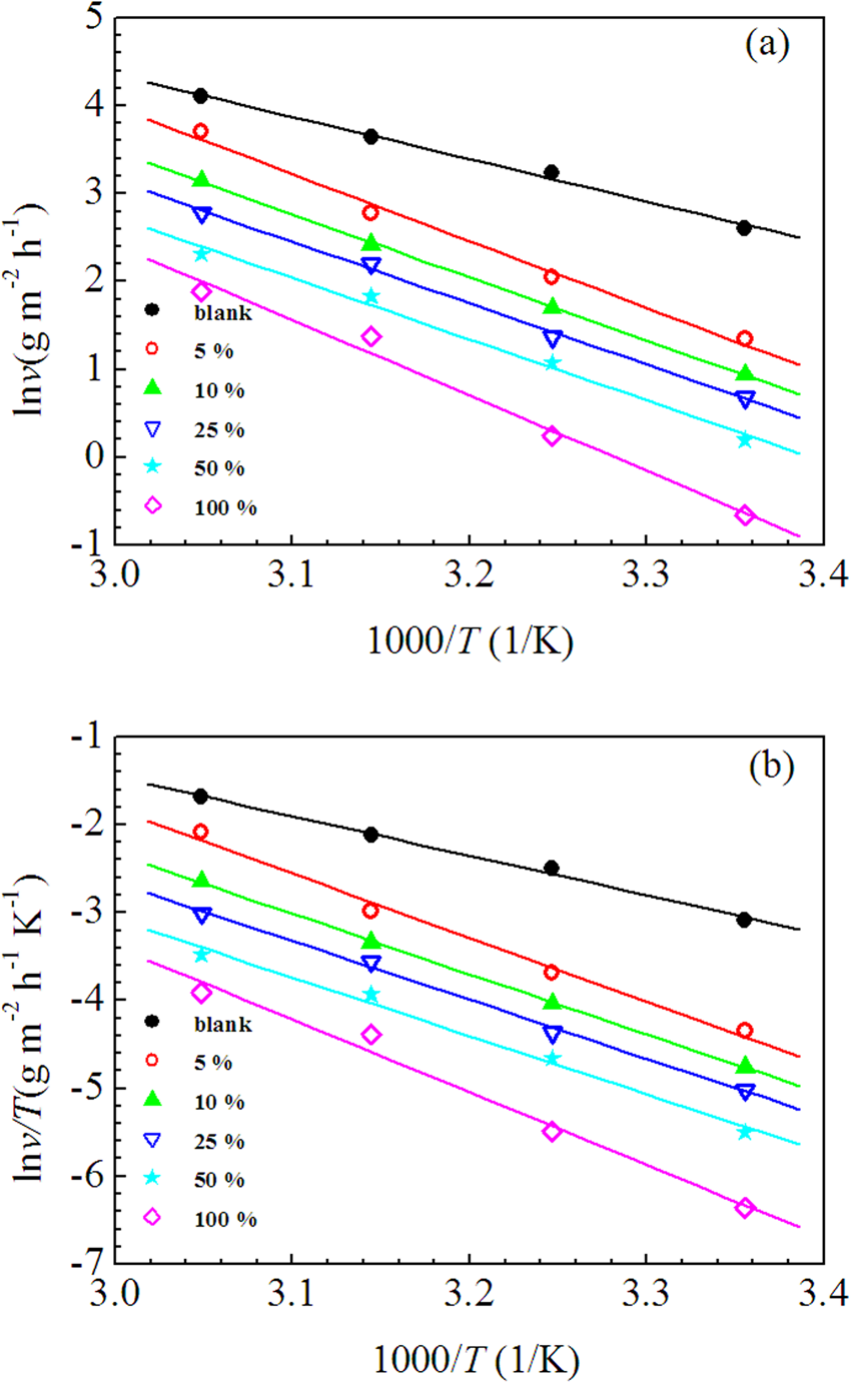
Arrhenius plots (**a**) and Transition state plots (**b**) for mild steel in 0.5 M H_2_SO_4_ solution in the absence and presence of various V/V concentrations of LLE.

Temperature is an important factor affecting the performance of inhibitor, and Table 3 indicates that the inhibition efficiency of LLE decreased with rising of temperature. To further understand the inhibitive mechanism of LLE, Arrhenius equation and transition state equation were employed to fit the corrosion rates at different temperatures. The two equations can be expressed respectively as follows^61,62^:

6 $$\mathrm{ln}\,\nu =-\frac{{{E}}_{{a}}}{{RT}}+\,\mathrm{ln}\,{A}$$

7 $$\mathrm{ln}\,\frac{\nu }{{T}}=\,\mathrm{ln}\,\frac{{R}}{{Nh}}+\frac{{\rm{\Delta }}{{S}}_{{a}}}{{R}}-\frac{{\rm{\Delta }}{{H}}_{{a}}}{{RT}}$$

where *v* is the corrosion rate obtained from weight loss measurements, *A* is the pre-exponential factor, *h* is the Plank’s constant, *N* is the Avogadro’s number, *E*_a_ is the apparent activation energy, Δ*S*_a_ is the apparent entropy of activation, Δ*H*_a_ is the apparent enthalpy of activation. Correspondingly, the fitting results are shown in Figure 7, the plots in Figure 7 present straight lines, thus according to the slopes and intercepts of these straight lines, the kinetic parameters including *E*_a_, Δ*H*_a_ and Δ*S*_a_, are calculated and listed in Table 6. The value of *E*_a_ is greater in 0.5 M H_2_SO_4_ solution containing LLE than that in blank solution, which indicate that the adsorption of LLE on the mild steel surface leads to the dissolution of steel become difficult. And just as expected, the values of *E*_a_ and Δ*H*_a_ follow the same trend of variation with the concentration of LLE, according to the transition state theory, Δ*H*_a_ = *E*_a_ −*RT* ^64^. Additionally, Table 6 shows that the values of Δ*S*_a_ is more positive in inhibited solution compared to that in blank solution, which indicate the system disorder increased from reactant to activated complex during the corrosion process^48,65^.

**Table 6:** Activation p.arameters for mild steel in 0.5 M H_2_SO_4_ solution in the absence and presence of various V/V concentrations of LLE.

*c*(% V/V)	*E*_a_(kJ/mol)	Δ*H*_a_(kJ/mol)	Δ*S*_a_(J K^−1^ mol^−1^)
blank	39.96	37.36	−97.55
5	63.21	60.62	−30.87
10	59.55	56.96	−46.04
25	58.08	55.48	−53.19
50	57.85	55.25	−57.33
100	71.25	68.65	−19.76

**Figure 8:**
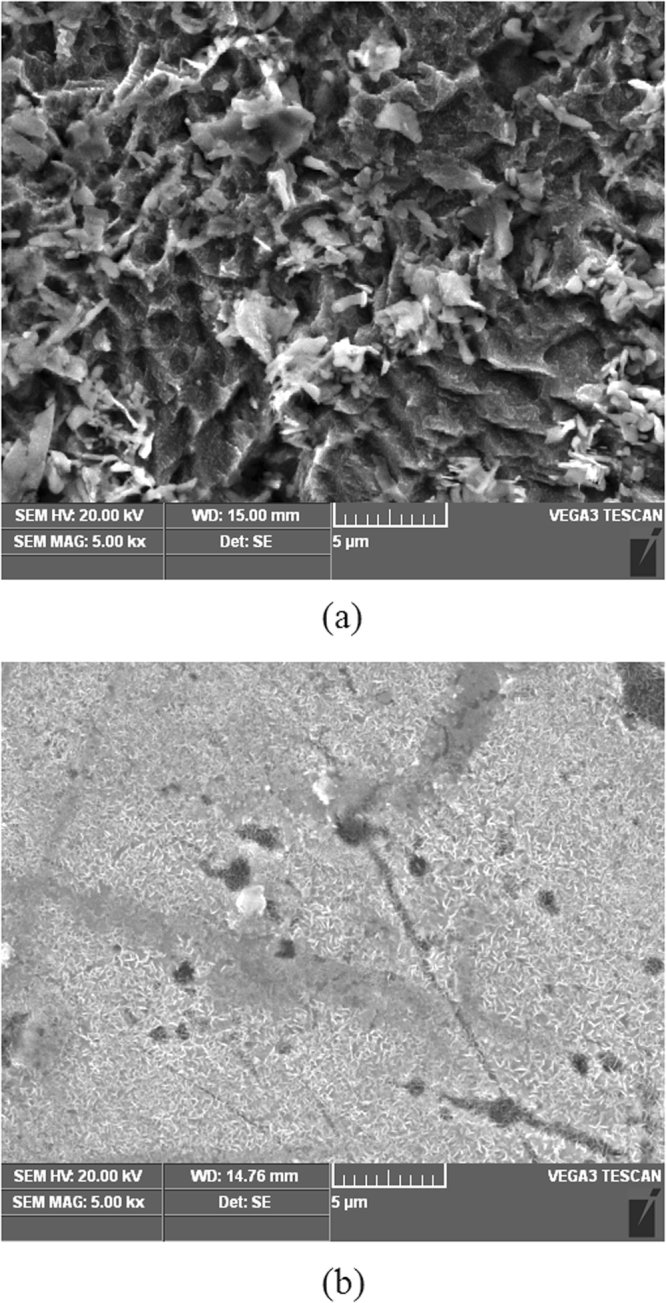
SEM micrographs of mild steel surface after immersed in 0.5 M H_2_SO_4_ solution in the absence of LLE (**a**) and in presence of 100% V/V LLE (**b**) for 4 h at 298 K.

The SEM micrographs of mild steel samples after immersed in blank solution and in inhibited solutions for 4 h at 298 K are shown in Figure 8. As shown in Figure 8a, in the absence of LLE, the mild steel surface is fairly rough and seriously damaged by the aggressive solution. In comparison, in presence of 100% V/V LLE, the steel surface is lightly damaged, which indicate the addition of LLE can effectively inhibit the corrosion of mild steel in the aggressive H_2_SO_4_ solution.

**Figure 9:**
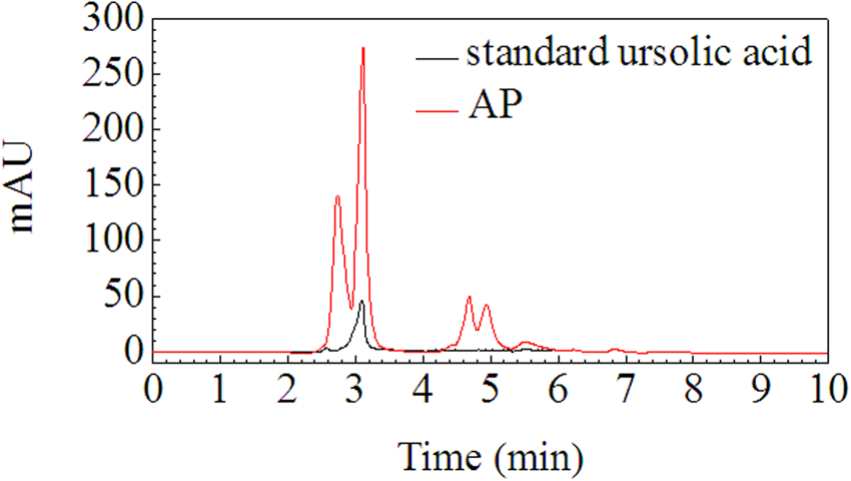
HPLC chromatograms of standard ursolic acid and AP.

**Figure 10:**
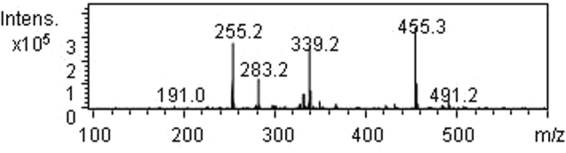
MS of AP.

**Figure 11:**
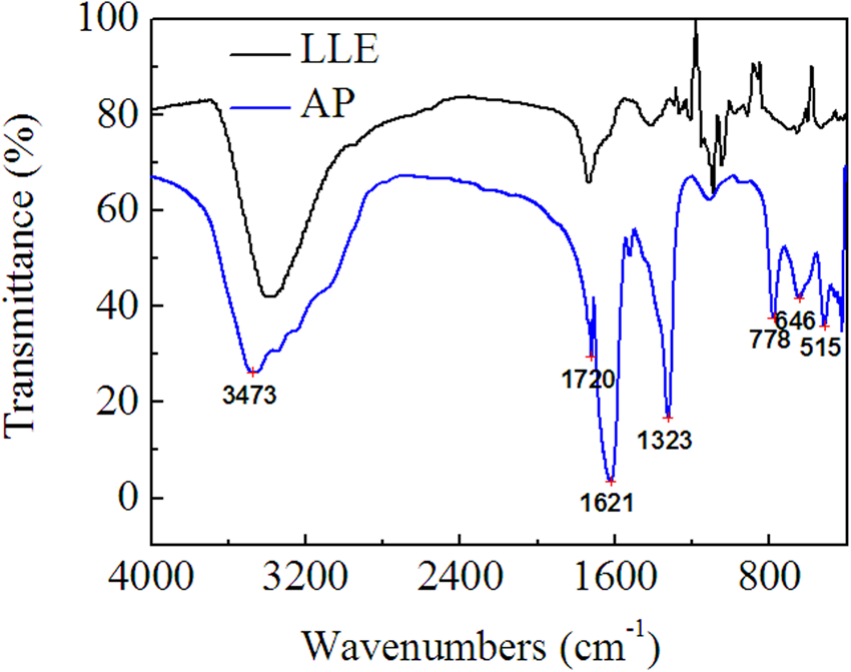
FTIR spectra of AP and the differential infrared spectroscopy of LLE.

**Figure 12:**
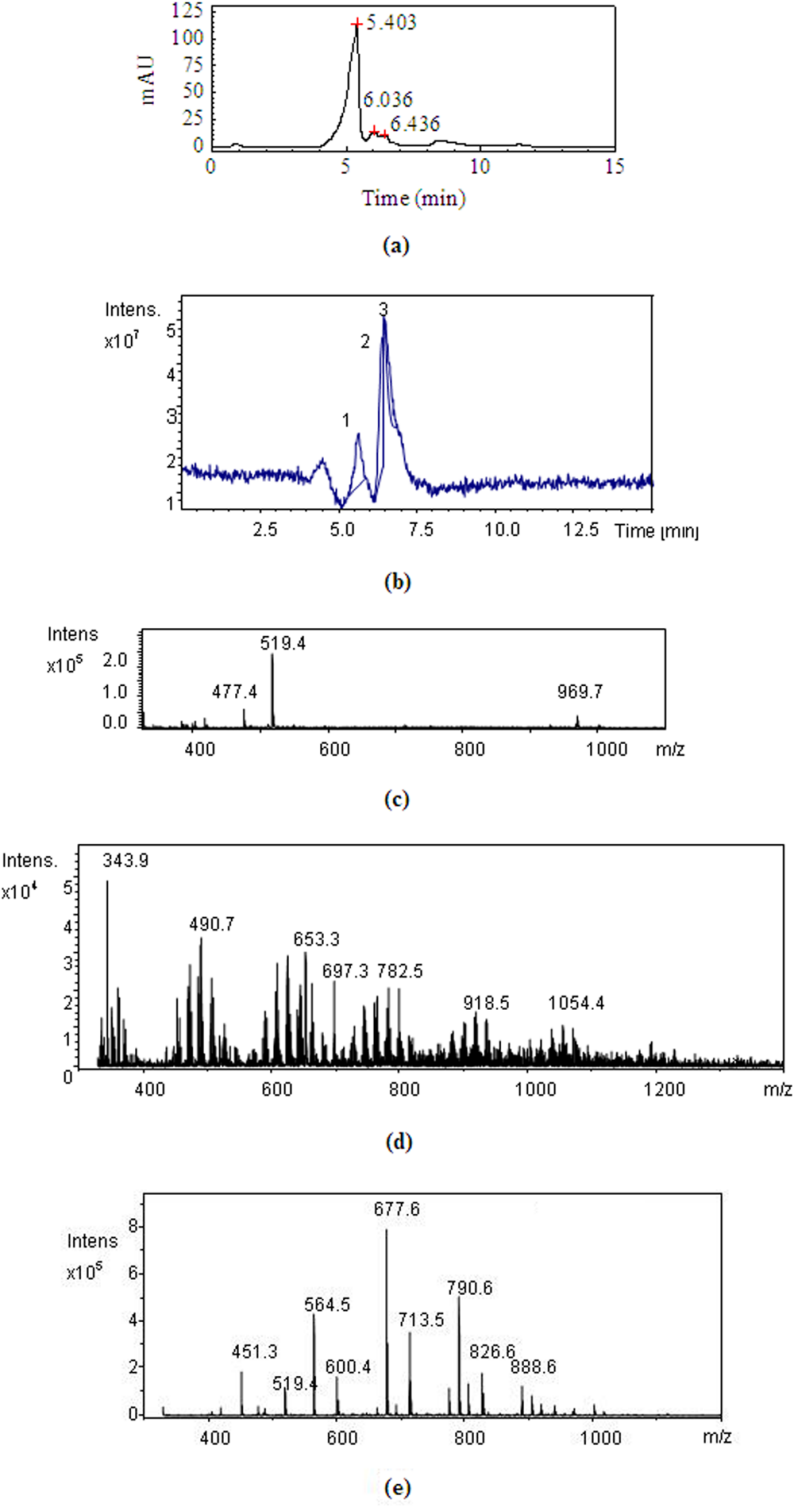
HPLC chromatograms of BP (**a**), total MS of BP (**b**), MS of BP at 4.3–4.7 min (**c**), MS of BP at 5.3–5.9 min (**d**) and MS of BP at 6.1–6.4 min (**e**).

**Figure 13:**
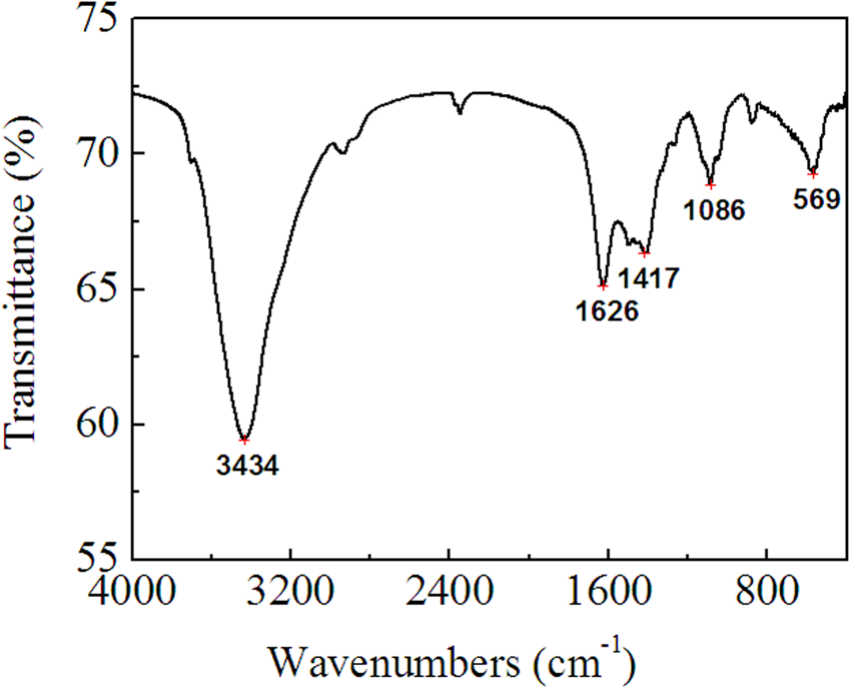
FTIR spectra of BP.

By pH-gradient sedimentation, LLE was preliminarily separated into acidic precipitate (AP), alkali precipitate (BP) and remaining solution (RS). HPLC chromatograms of standard ursolic acid and AP are shown in Figure 9, which indicate that ursolic acid is present in AP, but AP is not pure substance. This result is further confirmed by the MS of AP shown in Figure 10, in which, the quasimolecular ions [M-H]^−^ at *m/z* 191.0, 255.2 and 455.3 are in agreement with citric acid^66^, palmitic acid^67^ and ursolic acid^67,68^. Figure 11 shows the FTIR spectra of AP and the differential infrared spectra of LLE. The differential spectroscopy was obtained through the FTIR spectra of stock solution of LLE and the sulfuric acid solution using software OMNIC 7.0 by differential spectroscopic analysis. It is clearly seen that the infrared absorption peaks of AP are basically in accordance with those of LLE, indicating that AP is the main component of the LLE. Combined with the results of MS, the strong and broad peak at 3473 cm^−1^ can be attributed to the stretching vibrations of hydroxyl (OH) or carboxyl (COOH) groups, the peaks at 1720 cm^−1^ and 1621 cm^−1^ are the characteristic peaks of O = C and C = C, respectively^69,70^. Absorption peak at 1323 cm^−1^ may be indicate C-H bending vibration^69^, and the peaks at 778, 646, 515 cm^−1^ could be assigned to C-H of aliphatic and aromatic carbon. Figure 12 shows the HPLC chromatogram and MS of BP, then comparing mass spectrometric data with literatures, the peak at *m/z* 477.4 was consistent with the presence of isorhamnetin 3-O-galactoside or isorhamnetin-3-O-glucoside^43^, while the molecular ion at *m/z* 451.3 was identified as cinchonain Ia^69^ or cinchonain Ib^71,72^. The peak with [M-H]^−^ at *m/z* 519.4 probably produced by lignan of (+)-Pinoresinol-O-β-D-glucopyranoside^73^ or dehydrodiconiferylalcohol-9′-O-β-D-glucopyranoside^74^. The FTIR of BP is shown in Figure 13, the absorption peaks of 3434 and 1626 cm^−1^ are attributed to the stretching vibrations of hydroxyl and C = C, respectively^69,70^, the peak at 1417 cm^−1^ can be assigned to the bending vibrations of C-H or O-H^31^, the adsorption band 1473 cm^−1^ corresponds to the stretching vibration of C-O^69^, and the peak 569 cm^−1^ may be assigned to C-H of aliphatic and aromatic carbon. Consequently, the infrared absorption of BP is corresponding to the analysis of MS. Seven acids, including valine, tyrosine, phenylalanine, lysine, histidine and arginine were detected in RS, similar results were also reported by Yokota *et al*.^75^. Moreover, the color of RS changed with the pH value of the solution, which implies that RS contained anthraquinones.

**Figure 14:**
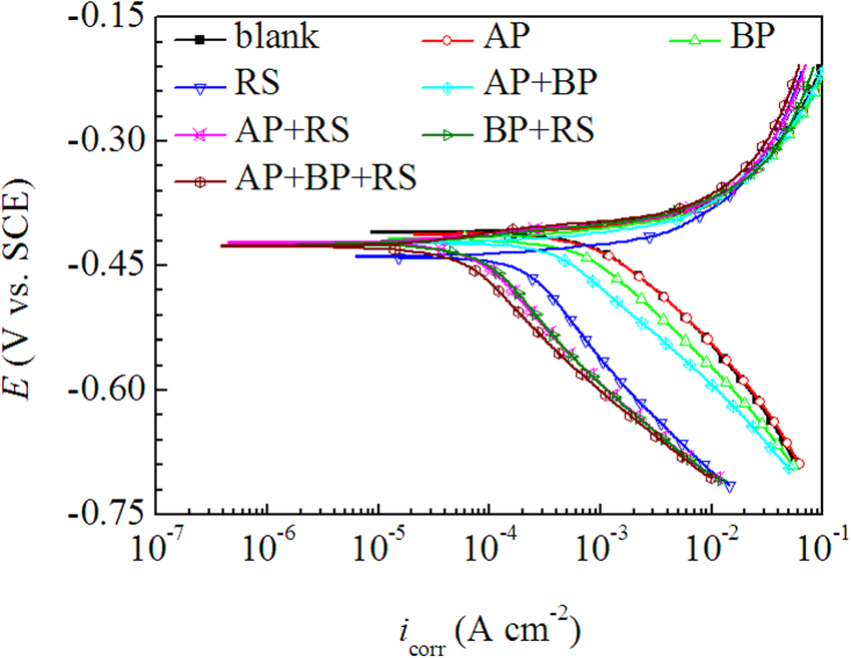
Potentiodynamic polarization curves for mild steel in 0.5 M H_2_SO_4_ solution without and with different isolates of LLE at 298 K.

Furthermore, the inhibition performance of the isolates and their mixtures was evaluated with the V/V concentration of 50% as a reference, the corresponding polarization curves and electrochemical parameters are shown in Figure 14 and Table 7, respectively. The results showed that AP can not inhibit the corrosion of mild steel in H_2_SO_4_ solution, and the inhibitory effect of BP was very weak, however, RS exhibited obvious corrosion inhibition. It is particularly important to note that the inhibition efficiency increased significantly when the isolate was mixed, and the inhibition efficiency of the mixture of three isolates is as high as 91%, which indicate that there is a strong synergistic effect between the chemical components of LLE.

**Table 7:** The electrochemical parameters for mild steel in 0.5 mol/L H_2_SO_4_ solution without and with different isolates of LLE at 298 K.

*c*(% V/V)	*E*_corr_ (V vs. SCE)	*I*_corr_(mA cm^−2^)	*η*(%)
blank	−0.409	0.611	\
AP	−0.412	0.676	\
BP	−0.417	0.589	3.7
RS	−0.432	0.204	66.7
AP + BP	−0.422	0.391	36.1
AP + RS	−0.422	0.070	88.6
BP + RS	−0.424	0.073	88.0
AP + BP + RS	−0.427	0.050	91.8

From the foregoing, the inhibition mechanism of LLE can be described as follows:

When the loquat leaves were immersed in H_2_SO_4_ solution, the organic compounds in the leaves, such as amino acids, organic acids, glycosides, and so on, were dissolved into the solution and were further protonated in strongly acidic solution. It is usually considered that the steel surface charges positive charge in acid solution^76,77^, which means that the protonated substances are difficult to adsorb on the steel surface due to the electrostatic repulsion, but Solmaz’s study found that the steel surface carries negative charge in HCl solution in the presence of inhibitors^78,79^, which allows the positively charged inhibitor molecules directly adsorb on the steel surface through electrostatic interaction, and then reduce the steel dissolution. However, Figure 2 showed a weak inhibitory effect of LLE on the anodic dissolution of iron, which implies that the steel surface in the investigated solutions is positively charged or has very little negative charge. These protonated substances also can adsorb on cathodic sites of the steel in competition with hydrogen ions, maybe due to the higher adsorption rate of the positively charged inhibitor molecules than that of hydrogen ions, thus the cathodic reaction of hydrogen ions reduction can be inhibited^78,79^, and the inhibitive effect of LLE on the cathodic reaction was clearly shown by polarization curves in Figure 2. As a result, LLE act as a modest cathodic inhibitor.

## Methods

### Electrolyte and Material

The corrosive solution 0.5 M H_2_SO_4_ was prepared using analytical grade sulphuric acid and distilled water. The chemical composition (in wt%) of mild steel as follows: C (0.16%), Si (0.18%), Mn (0.29%), P (0.014%), S (0.013%) and Fe for balance, and the preparation of specimens used in the experiments were the same as reported earlier^48–50^. Before conducting the test, the samples were mechanically abraded with emery paper up to 800 grit. During the experiment, the test solution was open to the air and under a static condition, meanwhile, temperature was controlled by a water thermostat with an accuracy of 1 K.

### Preparation of Loquat Leaves Extract

Loquat leaves were picked up at the campus of Sichuan University of Science & Engineering in December 2014, and then rinsed well under running water before drying. The leaves were then kept in an oven at 50 °C for 72 h and ground to powder. And the powder was stored in a sealed container. Afterwards, the stock solution of loquat leaves extract (LLE) was prepared as follows: 100 g of powdered leaves were soaked in 1 L 0.5 M H_2_SO_4_ solutions for 12 h before the extract solution was filtered and stored. After that, the test solution with inhibitor, i.e. 5, 10, 25, 50 and 100% (V/V), were prepared through diluting the stock solution with corresponding amount of 0.5 M H_2_SO_4_ solution.

### Isolation and characterization of LLE

LLE was first neutralized with sodium hydroxide, when the pH value of LLE increased to 1, white substance started to precipitate out, continuously adding alkali solution until before the color of LLE changes dramatically, at this point, the pH value of solution is about 5, after standing for 24 hours, the acidic precipitate of LLE (AP) was separated by centrifugation, then AP was washed three times with distilled water and dried in vacuum. While the remaining solution was continued alkalization, at pH 12, brown precipitate was found. The pH value of solution was adjusted to close to 14 in order to make precipitation more complete. Then alkali precipitation (BP) was obtained by centrifugation after the solution was stood for 24 h, and the remaining solution (RS) was neutralized with sulfuric acid and was not further isolated.

The methanol solution of AP was analyzed by Agilent 1100 high performance liquid chromatograph (HPLC) used ursolic acid as the reference substance, in which, the analytical column used was phenomenex C18, the mobile phase was methanol aqueous solution (90: 10, V/V) with a flow rate of 0.5 mL/min at 30 °C, and eluent was detected at 215 nm. Moreover, the composition of AP was further confirmed by mass spectrometry (MS). The composition of BP was detected by liquid chromatography-mass spectrometry (LC-MS) using Agilent LC1100/MSD-VL, and the chromatographic conditions were consistent with the foregoing. Furthermore, Hitachi L-8900 amino acid analyzer was employed to determine amino acids in RS, and FTIR spectra (KBr) was measured by Nicolet-6700 spectrophotometer.

### Measurement Method

Electrochemical measurements were conducted in a three-electrode cell assembly with mild steel working electrode, saturated calomel electrode (SCE) and platinum electrode using CHI660E electrochemical work station (Shanghai Chenhua Instruments Co. Ltd., China). All potential data reported were referred to SCE reference electrode. The working electrode was initially immersed into test solution at OCP until it reached steady state. Then, EIS was measured at OCP. Ultimately, the polarization curve was obtained by potentiodynamic scanning method. The procedure for electrochemical measurements had been previously described in detail^48–50^.

Weight loss measurements were performed in a wild-mouth glass bottle containing 500 mL of test solution for 4 h at different temperatures (298, 308, 318 and 328 K). After that, the microscopic examination of the specimen surface was performed using a Tescan Vega3 scanning electron microscopy at high vacuum. The experimental procedures and data processing were similar to previously reported researches^48–50^.

## Conclusions

1. LLE can be considered as a modest cathodic inhibitor which mainly inhibits the cathodic process on the corrosion of mild steel in 0.5 M H_2_SO_4_ solution, and its inhibitive efficiency increases with the increment of LLE concentration, but decreases when rising temperature.
2. In the presence of LLE, the charge transfer resistance increases while double layer capacitance declines, confirming the adsorption of inhibitor molecules on the mild steel surface. The adsorption of LLE on steel surface is an exothermic process and obeys Langmuir adsorption isotherm.
3. The corrosion inhibition of LLE is caused by the combination of various components in the extract, and there is a synergistic effect between the components.

### Data Availability

Te datasets generated during and/or analyzed during the current study are available from the corresponding author upon reasonable request.
